# Inhibition of the extracellular signal‐regulated kinase pathway reduces the inflammatory component in nucleus pulposus cells

**DOI:** 10.1002/jor.25273

**Published:** 2022-02-01

**Authors:** Adel Tekari, Alessandro Marazza, Katherine Crump, Paola Bermudez‐Lekerika, Benjamin Gantenbein

**Affiliations:** ^1^ Tissue Engineering for Orthopaedics and Mechanobiology, Bone and Joint Program, Department for BioMedical Research (DBMR), Medical Faculty University of Bern Bern Switzerland; ^2^ Laboratory of Molecular and Cellular Screening Processes, Centre of Biotechnology of Sfax University of Sfax Sfax Tunisia; ^3^ Alzheimer's Center at Temple, Lewis Katz School of Medicine Temple University Philadelphia Pennsylvania USA; ^4^ Department of Orthopaedic Surgery and Traumatology, Inselspital, Bern University Hospital University of Bern Bern Switzerland

**Keywords:** arthrosis, ERK, intervertebral disc, nucleus pulposus, TNF‐α

## Abstract

Intervertebral disc (IVD) degeneration is a spinal disorder that triggers an inflammatory response and subsequent development of spinal pseudoarthrosis. The aim of the present study is to elucidate the role of the extracellular signal‐regulated kinase (ERK) pathway in inflammation‐induced IVD cells. Inflammatory human nucleus pulposus (NP) cells (NPCs) were induced using tumor necrosis factor‐α and the ERK pathway was blocked using a selective molecule‐based inhibitor U0126. Gene expression of catabolic and anabolic markers, proinflammatory, and NPCs markers was investigated. The enzymatic activity of matrix metalloproteinases (MMP)2/MMP9 was determined by gelatin zymography and nitrite production was assessed by Griess reaction. The NPC metabolic activity and viability were assessed using resazurin sodium‐salt and live/dead assays, and subsequently, the specificity of U0126 on ERK1/2 signaling was determined. The catabolic enzyme MMP3 (*p* = 0.0001) and proinflammatory cytokine interleukin 6 (*p* = 0.036) were downregulated by U0126 in NPCs under inflammatory conditions. A significant increase of the cytokeratin 19 (*p* = 0.0031) was observed, suggesting a partial and possible recovery of the NP phenotype. U0126 does not seem to have an effect on prostaglandin production, aggrecanases, or other anabolic genes. We confirmed that U0126 selectively blocks the ERK phosphorylation and only affects the cell metabolic activity without the reduction of viable cells. Inhibition of ERK signaling downregulates important metalloproteinases and proinflammatory cytokines, and upregulates some NP markers, suggesting its potential to treat IVD degeneration.

## Graphical abstract

Matrix metalloproteinases 3 (MMP3), interleukin 6, and nitric oxide were downregulated by U0126 in tumor necrosis factor‐α (TNF‐α)‐induced nucleus pulposus (NP) cells. A significant increase of KRT19 and random modulation of ACAN and COL2 suggest a partial recovery of the NP phenotype. U0126 selectively blocks extracellular signal‐regulated kinase (ERK) phosphorylation and only affects the cell metabolic activity without reduction of viable cells. ERK pathway is implicated in NP inflammatory processes and ERK1/2 inhibition could provide some protection against the adverse effects of TNF‐α in the tissue.

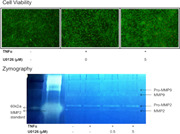

## 1 INTRODUCTION

Low back pain affects millions of people in industrialized societies every year, making it a leading cause of disability with significant economic and social burdens.^1^, ^2^, ^3^ Chronic low back pain is strongly associated with intervertebral disc (IVD) degeneration which results in increasing pain during the execution of daily spinal movements. The IVD consists of an inner nucleus pulposus (NP) surrounded by the annulus fibrosus (AF) tissue, and hyaline articular cartilage that is located at the endplates between the IVD and two adjacent vertebral bodies. The NP is a gelatinous‐like and avascular connective tissue containing a highly organized extracellular matrix (ECM) rich in proteoglycans and collagens with few numbers of cells. The nucleus pulposus cells (NPCs) actively regulate homeostasis in the ECM by several growth factors and cytokines through autocrine and paracrine signals. The early stage of disc degeneration is often asymptomatic^4^ and chronic inflammation tends to develop within the spine. This produces high stresses in the disc tissue, which begins with the synthesis of catabolic enzymes,^5^ resulting in degradation and loss of the ECM structures present in the IVD. The loss of IVD structures leads to leakage of the connective tissue from the NP through the AF, which causes compression of peripheral nerves, resulting in pain and limited spinal movements.

Noninvasive interventions including medications, steroid injection, and physical therapy are recommended most often but have limited long‐term efficacy^6^, ^7^ since they do not maintain or restore the native tissue structure in degenerative discs. Current surgical treatments aim to eliminate discogenic pain by replacing the injured tissue with a functional biological substitute or prosthesis. However, surgery for disc degeneration also yielded mixed clinical outcomes and often results in incomplete interbody fusion.^8^

Interleukin 1β (IL1β) and tumor necrosis factor‐α (TNF‐α) are proinflammatory cytokines known to be key mediators in the development and progression of disc degeneration and low back pain.^5^ Many studies have shown that TNF‐α is highly expressed in degenerative IVD tissues^9^, ^10^ and surgical samples obtained from patients with a history of low back pain revealed higher levels of TNF‐α‐positive cells than autopsies from healthy controls.^11^ TNF‐α causes an upregulation of ECM‐degrading enzymes and decreases the expression of matrix‐specific matrix proteins.^12^ Thus, identifying and targeting signaling pathways responsible for the TNF‐α‐mediated inflammation could be a promising therapeutic option for IVD tissue regeneration.

Extracellular signal‐regulated kinase (ERK), a downstream signal of the mitogen‐activated protein kinase (MAPK) signaling cascade, is an important inflammatory pathway that plays a critical role in the production of inflammatory cytokines and the activation of procatabolic responses induced by TNF‐α in chondrocyte‐lineage cells.^13^ Inhibition of MAPK signaling attenuates the decrease of collagen type II (COL2A1) and aggrecan (ACAN) without inducing apoptosis in primary rats and immortalized chondrocytes.^14^ In agreement with this, inhibition of MAPK/ERK activity enhances chondrogenesis of mesenchymes,^15^ and TNF‐α‐induced nuclear factor‐κB (NF‐kB) DNA binding in chondrocytes is reduced by inhibition of MAPK signaling.^14^ Recent studies suggest ERK is a catabolic pathway in the degeneration of IVD. In AF cells, ERK was shown to mediate IL1‐induced upregulation of cyclooxygenase (COX),^16^ important aggrecanases, and metalloproteinases,^17^ while ERK inhibition decreased IL1β‐induced apoptosis.^18^

Nevertheless, the role of the ERK pathway in effecting the action of TNF‐α in NPCs has not been fully elucidated. Hence, the aim of the present study is to clarify the role of ERK in inflammation‐induced human NPCs culture, a widely accepted in vitro model and approach to study spinal degeneration and regeneration.

## 2 METHODS

### 2.1 Human NP isolation and culture

Human IVD was collected from trauma patients undergoing spinal fusion surgery without any history of disc degeneration before the operation. The procedure was performed with patients’ written consent and was approved by the local ethical committee of the canton of Bern, Switzerland. The disc materials were collected from patients (Table 1) aged between 18 and 59 years old (34.9 ± 17.9 [mean ± SD]). The NP tissue was separated from the outer AF of the disc by an experienced surgeon and subsequently processed within 24 h after the surgery. Data were anonymized and only the sex and age were recorded from each donor. The NPCs were isolated by sequential digestion of the NP tissue fragments with 1.9 mg/ml pronase (Roche) for 1 h followed by collagenase II (Worthington) at 37°C overnight on a plate shaker. The remaining undigested NP tissue debris was removed by filtration through a 100 μm cell strainer (Falcon; Becton Dickinson) and cell viability was determined by Trypan blue exclusion.

**Table 1:** Patient donor list of nucleus pulposus tissue samples enrolled in the study

Donor ID	Level	Age	Sex	Passage number	Method
hIVD1	L3–L4	33	M	P3	qPCR, RSSA, NO
hIVD2	L3–L4	51	M	P3	qPCR, RSSA, NO
hIVD3	L1–L2	20	M	P2	qPCR
hIVD4	T12–L1	50	F	P3	qPCR
hIVD5	L1–L2	18	M	P2	qPCR, RSSA, Zymo, WB
hIVD6	L2–L3	18	M	P3	qPCR
hIVD7	T12–L4	23	F	P2	RSSA
hIVD8	T12–L1	59	F	P2	RSSA
hIVD9	C5–C6	18	M	P3	LD
hIVD10	C7/T1	59	M	P4	LD

*Note*: Nucleus pulposus tissues were collected from posttraumatic intervertebral disc subjects without a history of disc degeneration and includes a total of 10 patients identified with their ID number, surgery location within the spine (level), age, sex, passage number, and the purpose of the investigation (molecular method).

Abbreviations: C, cervical; F, female; hIVD, human intervertebral disc; L, lumbar; LD, live/dead assay; M, male; NO, nitric oxide; qPCR, quantitative polymerase chain reaction; RSSA, resazurin sodium salt assay; T, thoracic; WB, Western blot; Zymo, gelatin zymography.

The NPCs were expanded in proliferation medium (Dulbecco's modified Eagle's medium [Sigma‐Aldrich] containing 10% fetal bovine serum [Sigma‐Aldrich] and penicillin/streptomycin [100 U/ml and 100 μg/ml, respectively; Merck]) until confluency. To amplify the low number of NPCs obtained after digestion of the biopsy, the NPCs were expanded in the proliferation medium until reaching several passages and a cell stock was cryopreserved for further analysis. Low‐passage (less than 3 passages) NPCs were used in this study.

### 2.2 Induction of proinflammatory environment and ERK inhibition

NPCs were seeded at a density of 5 × 10^4^ cells/well in 24‐well plates in the proliferation medium and left overnight for cell adherence. Induction of a proinflammatory microenvironment in NPCs was performed by addition of 10 ng/ml of human recombinant TNF‐α (Peprotech) to the wells as previously described.^19^, ^20^, ^21^ Control cultures represented NPCs with (positive control) or without (negative control) TNF‐α.

Alternatively, the cells were pretreated with a selective molecule‐based inhibitor of ERK, U0126 (Selleck Chemicals) at 0.5 and 5 µM for 1 h to inhibit activation of the ERK pathway and subsequently stimulated with TNF‐α (10 ng/ml). The cultures were maintained for 3 days and collected thereafter for downstream applications.

### 2.3 NPC metabolic activity

The NPCs were seeded at a density of 2 × 10^3^ cells/well in 96‐well plates in the proliferation medium and left overnight for cell adherence. To address whether blocking the ERK pathway or a combination of TNF‐α and ERK inhibition may reduce cell metabolism, NPCs were stimulated with 10 ng/ml TNF‐α and increasing concentration of a U0126 ranging from 0.1 to 10 µM after a pretreatment of 1 h. The cultures were collected after 3 days and cell metabolic activity was determined with a resazurin red solution (Sigma‐Aldrich) as previously described.^22^ Briefly, the cultures were incubated with 50 µM resazurin red solution in a humidified atmosphere (5% CO_2_, 37°C) for 2 h. The absorbance was measured at 580 nm on a microplate reader (SpectraMax M5; Bucher Biotec).

### 2.4 NPC viability assay

The NPCs were seeded at a density of 2 × 10^4^ cells/well in 24‐well plates and stimulated with 10 ng/ml TNF‐α and increasing concentration of U0126 ranging from 0.1 to 10 µM after a pretreatment of 1 h as indicated. The NPCs cultures were maintained thereafter in the proliferation medium with supplements for three days. Positive control samples were NPCs grown for 2 weeks (until confluence) and treated with 1 M sodium chloride (NaCl) or 70% ethanol (EtoH) for 20 min. To test the living and dead cells, NPCs were stained with 1 µM calcein‐AM and 1 µM ethidium homodimer‐1 in a serum‐free medium for 20 min at 37°C. Cell fluorescence was monitored on an inverse microscope (Leica; DM IL; filters: I3 S 450–490 nm and N2.1 S 515–560 nm). High‐resolution digital photos were then taken with both filters separately (2 vision fields/well).

### 2.5 Analysis of gene expression

Total RNA was extracted as previously described^23^ from the NPCs after stimulation with TNF‐α and inhibition of the ERK signaling. Total RNA was purified with a DNA Digestion Kit (Sigma‐Aldrich) and reverse transcription was performed with iScript cDNA Synthesis Kit (Bio‐Rad).^24^ NP‐specific markers, including ACAN, COL2A1, and cytokeratin 19 (KRT19), anabolic markers, including insulin‐like growth factor 1 (IGF1) and transforming growth factor β1 (TGFβ1), catabolic markers, including matrix metalloproteinase 3 (MMP3), MMP13, and a disintegrin and metalloproteinase with thrombospondin motifs 5 (ADAMTS5), proinflammatory markers, including IL6 and COX2, and the ribosomal 18S RNA as reference gene, were determined. Human‐specific oligonucleotide primers (Table 2) (Microsynth) were designed with Beacon Designer^TM^ (Premier Biosoft) based on the sequences of the nucleotides from GenBank. The efficiency and melting curves of the amplicons were tested to determine specific amplification. Relative gene expression was determined by application of a threshold cycle and normalized to control NPCs using the $2^{‐∆∆C_{t}}$ method according to Livak and Schmittgen.^25^

**Table 2:** Custom‐designed DNA primers used in the real‐time qPCR study

Gene	Forward sequence	Reverse sequence
18S	CGA TGC GGC GGC GTT ATT C	TCT GTC AAT CCT GTC CGT GTC C
ACAN	TCT GGA GTA GAG GAC ATC	AGG AAG TTC ACT GAC ATC
COL2	AGC AGC AAG AGC AAG GAG AA	GTA GGA AGG TCA TCT GGA
KRT19	TCT TGC TGC TGA TGA CTT	CCT CTT CGT GGT TCT TCT
IGF1	CAG ACA GGC ATC GTG GAT	TGA CTT GGC AGG CTT GAG
TIMP1	TCA ACC AGA CCA CCT TAT ACC A	ATC CGC AGA CAC TCT CCA T
TGFβ1	CGT GCT AAT GGT GGA AAC	GCT CTG ATG TGT TGA AGA AC
MMP3	CAA GGC ATA GAG ACA ACA TAG A	GCA CAG CAA CAG TAG GAT
MMP13	AGT GGT GGT GAT GAA GAT	CTA AGG TGT TAT CGT CAA GTT
ADAMTS5	GCT GTG CTG TGA TTG AAG A	TGC TGG TAA GGA TGG AAG A
IL6	GCC ACT CAC CTC TTC AGA AC	GCA AGT CTC CTC ATT GAA TCC A
COX2	GTC TGG TGC CTG GTC TGA	GTC TGG AAC AAC TGC TCA TCA C

*Note*: Amplicons were generated using a two‐step amplification cycling (95°C for 15 s and 57°C for 30 s for 45 cycles) and SYBR‐green master mix.

Abbreviation: qPCR, quantitative polymerase chain reaction.

### 2.6 Gelatin zymography

A gel zymography was performed in cell lysates after treatment of NPCs with TNF‐α and/or inhibition of the ERK signaling pathway to analyze the pattern of MMP2/MMP9 in different treatment groups. Briefly, the cell layers were washed with phosphate‐buffered saline (PBS) and homogenized with 0.5% Triton‐X (Sigma‐Aldrich) containing a protease inhibitor (Sigma‐Aldrich). The extracts were applied to a gelatin‐containing 10% sodium dodecyl sulfate‐polyacrylamide gel electrophoresis (SDS‐PAGE) and the gel was incubated in a developing buffer composed of 50 mM Trizma Base and 5 mM CaCl_2_ (pH 8.0) overnight at 37°C. Subsequently, the polyacrylamide gel was stained with 0.1% Coomassie blue R250 solution (Sigma‐Aldrich) for 2 h.

### 2.7 Nitric oxide assay

The levels of nitric oxide (NO) production in the NPCs culture medium were determined as the concentration of its stable nitrite oxidation product using the Griess Reaction Reagent Kit (Promega) according to the manufacturer's instruction.

### 2.8 Western blot analysis

Primary human NPCs (2.5 × 10^4^ cells/well) were seeded into six‐well culture plates and grown for 1 week in the proliferation medium without additional factors to allow for cell adherence. The medium was changed and the cells were treated with either TNF‐α, a combination of TNF‐α and U0126 (0.1, 0.5, 1, and 5 μM), or left as controls in dimethyl sulfoxide. The NPCs were pretreated with various concentrations of U0126 for 1 h and subsequently, TNF‐α (10 ng/ml) was applied to the cultures to stimulate an inflammatory microenvironment. After 30 min of treatment, adherent cells were rinsed with PBS and immediately lysed in the cell lysis buffer (CelLytic; Sigma‐Aldrich) containing a protease inhibitor cocktail (25 µg/ml; Sigma‐Aldrich) and phosphatase inhibitor (1 mM NaF, 1 mM sodium orthovanadate, 4 mM sodium tartrate, 1.15 mM sodium molybdate, and 2 mM imidazole; Biotool). The total protein content in the cell layers was determined by a Bradford protein assay. The samples (10 μg) were subjected to 12% SDS‐PAGE and transferred to a polyvinylidene fluoride membrane. Nonspecific binding sites were blocked using 5% skim milk in Tris‐buffered saline with 0.05% Tween (TBST) for 1 h at room temperature. The membrane was washed several times with TBST followed by incubation with a rabbit anti‐phospho‐ERK1/2 (p‐ERK1/2) and/or ERK1/2 primary antibodies (Cell Signaling Technology) at 4°C overnight. Detection was achieved using a goat anti‐rabbit antibody (LI‐COR Biosciences GmbH), and the band intensity was quantified with Odyssey Software (LI‐COR).

### 2.9 Statistical analysis

Differences in a real‐time polymerase chain reaction, resazurin sodium‐salt assay, nitrite production, and Western blot were evaluated by a one‐way analysis of variance with Bonferroni's post hoc test using GraphPad Prism (version 6.0 f for Mac OS; GraphPad Software Inc). *p* < 0.05 was considered significant.

## 3 RESULTS

### 3.1 U0126 effect on the NPC metabolic activity and viability

**Figure 1:**
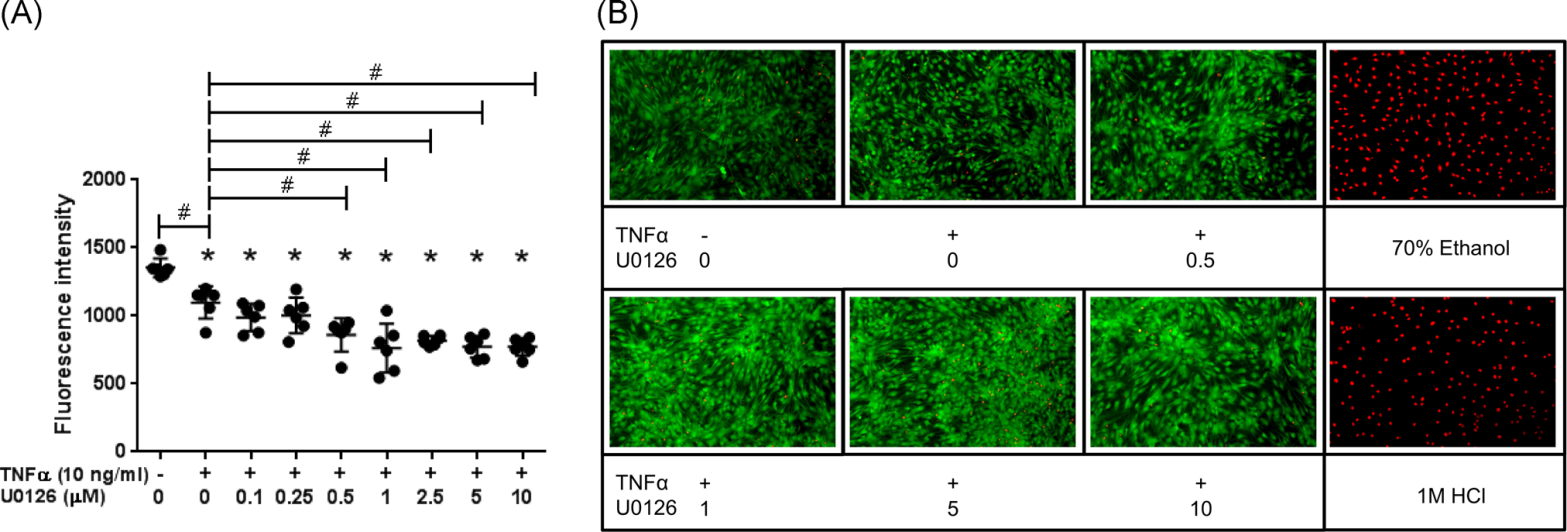
Cell metabolic activity (A) and viability (B) following treatment of NPCs with TNF‐α (10 ng/ml) and different concentrations of U0126 after 2 h of incubation with resazurin sodium‐salt assay or 20 min with labeling cells with calcein‐AM and ethidium homodimer‐1. A dose‐dependent reduction in metabolically active NPCs with increasing U0126 concentrations was observed. *p < 0.05 as compared to positive control (TNF‐α treatment) or ^#^ *p* < 0.05 as compared to TNF‐α‐treated NPCs. The number of viable NPCs (green labeling) remained almost the same in the different treatment groups with only a few dead cells (red labeling). NPCs treated with NaCl or EtoH served as controls and were all dead cells. NPC, nucleus pulposus cell; TNF‐α, tumor necrosis factor‐α [Color figure can be viewed at wileyonlinelibrary.com]

Cellular metabolic activity and viability were analyzed using the resazurin sodium‐salt assay and live/dead assay, respectively, to verify whether U0126 induces any effect on the proliferation and viability of the disc cells. There was a dose‐dependent decrease in the NPCs metabolic activity with increasing concentrations of U0126 compared to the control group. The NPC metabolic activity was significantly reduced (*p* = 0.0016, Figure 1A) by U0126 at 0.5 µM and beyond 1 µM (*p* < 0.0001). However, the viability of NPCs was almost unaffected in all treatment groups with only a few dead cells (Figure 1B). NPCs treated with 1 M sodium chloride (NaCl) or 70% EtoH served as dead cell controls (ethidium homodimer‐1‐positive cells, red labeling, Figure 1B). This data suggested that U0126 might block proliferation without affecting NPC viability at high concentrations (micromolar range). In addition, TNF‐α (10 ng/ml) alone significantly reduced (*p* = 0.001) the NPCs metabolic activity compared to untreated cells. The TNF‐α‐stimulated NPCs were morphologically different, spindle‐shaped with a fibroblast‐like appearance and less dense compared to untreated cells observed microscopically.

### 3.2 Gene expression

**Figure 2:**
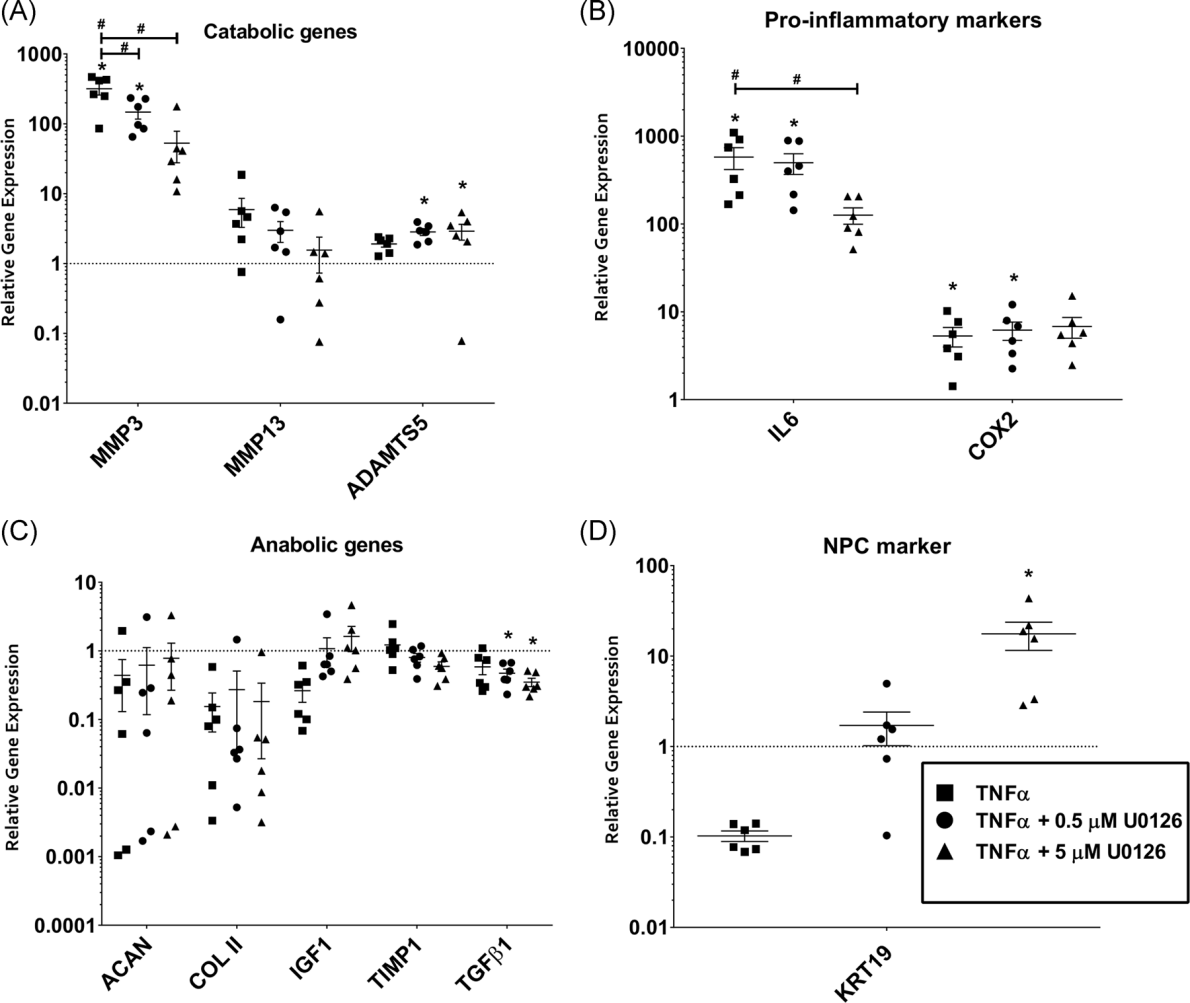
Modulation of human NPCs transcript levels induced by TNF‐α (10 ng/ml) with or without ERK inhibition (0.5 or 5 µM of U0126). The change of catabolic genes (A), proinflammatory markers (B), anabolic genes (C), and NPCs marker (D). Control values (without treatment) were set as 1 and different levels were normalized to their respective controls. *p < 0.05 as compared to untreated controls or ^#^ *p* < 0.05 as compared to TNF‐α‐treated groups. ERK, extracellular signal‐regulated kinase; IL6, interleukin 6; NPC, nucleus pulposus cell; TNF‐α, tumor necrosis factor‐α

The effect of TNF‐α stimulation followed by ERK inhibition was investigated at the transcript level of NPCs of catabolic, anabolic genes, and NPCs markers including matrix components, and anticatabolic, and inflammatory markers. The addition of TNF‐α (10 ng/ml) to the NPCs cultures resulted in increased expression of important catabolic and proinflammatory pathway markers including MMP3 (*p* < 0.0001, 317‐fold, Figure 2A), COX2 (5‐fold, Figure 2B), and IL6 (*p* = 0.002, 580‐fold). A nonsignificant modulation in the anabolic and NPCs markers, including ACAN (2‐fold, Figure 2C), COL2 (7‐fold), IGF1 (4‐fold), TGFβ1 (2‐fold), and KRT19 (10‐fold, Figure 2D) was observed after TNF‐α stimulation.

There was a random variation of COL2 (1.5‐fold and 1.8‐fold), and ACAN (1.8‐fold and 1.2‐fold) transcript levels following ERK inhibition by U0126 at 0.5 and 5 µM, respectively, in TNF‐α‐treated NPCs compared with the TNF‐α alone group (Figure 2C). These variations were only random with no trend towards a significant effect of ERK inhibition. Similarly, although IGF1 expression showed a 4‐fold and 6‐fold change following the addition of U0126 to TNF‐α‐treated cells at 0.5 and 5 µM, respectively, the change was not significant and, therefore, it cannot be concluded that ERK inhibition has any effect on IGF1 expression (Figure 2C). KRT19 was upregulated 17‐fold and 170‐fold while treating the TNF‐α NPCs cultures with U0126 at 0.5 and 5 µM, respectively (Figure 2D).

The catabolic marker MMP3 transcript levels were significantly reduced following treatment of the TNF‐α‐stimulated NPCs cultures with U0126 at 0.5 (*p* = 0.02) and 5 µM (*p* = 0.008). The MMP13 transcript levels were nonsignificantly decreased by 2‐fold (*p* = 0.72) and 4‐fold (*p* = 0.48) followi and 5 µM, respectively (Figure 2A). Similarly, the proinflammatory cytokine IL6 was significantly reduced by addition of U0126 at 0.5 (*p* = 0.02) and 5 µM (*p* = 0.01) to inflamed NPCs cultures (Figure 2B). The ADAMTS5 levels were significantly increased following treatment of NPCs with U0126 at 0.5 (*p* = 0.016) and 5 µM (*p* = 0.013). The COX2 messenger RNA (mRNA) levels remained almost the same in the U0126 treated cultures as compared to the TNF‐α alone group. Similarly, the TIMP and TGFβ1 levels showed similar random variation in treated groups compared to those of control groups.

### 3.3 Gelatinases expression and nitric oxide production

**Figure 3:**
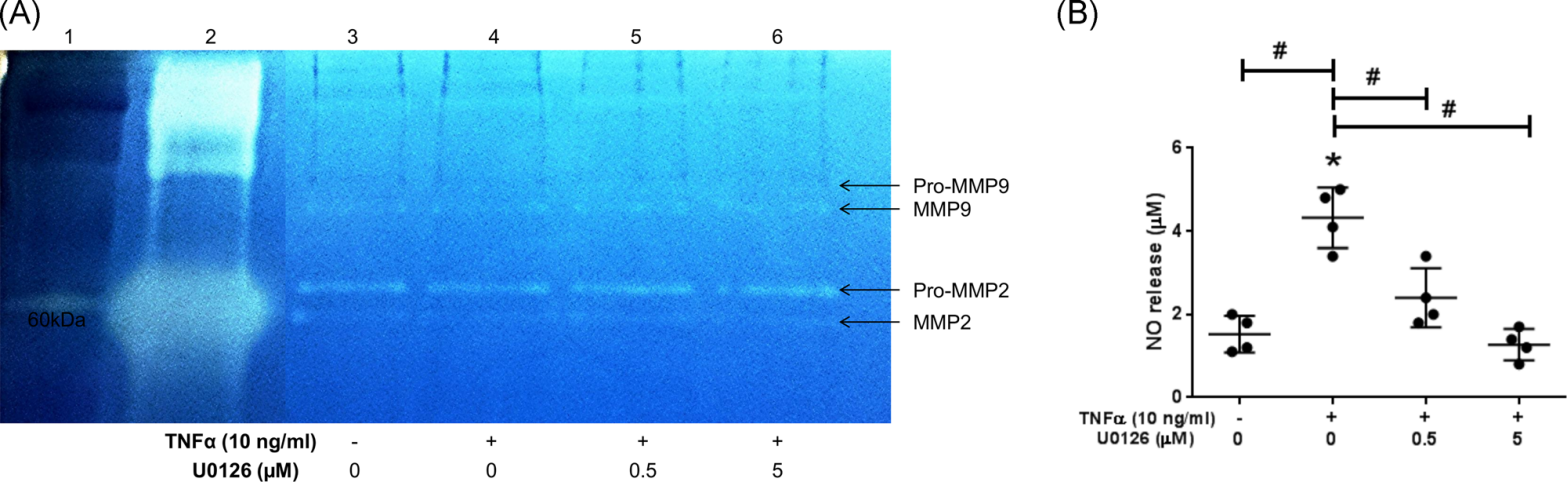
Gel zymography for the detection of the enzymatic activity of MMP2/MMP9 (A) and nitrite release (NO) in NPCs. (A) Lane 1: ladder with a visible band being 60 kDa, Lane 2: MMP2 standard at 100 ng/ml, Lane 3–6: treatment groups of NPCs with or without TNF‐α (10 ng/ml) and U0126. Pro‐ and active MMP2/MMP9 are indicated by arrows on the gels. (B) Nitrate release was detected in NPCs medium culture with TNF‐α and/or U0126 (0.5 or 5 µM). *p < 0.05 as compared to untreated controls or ^#^ *p* < 0.05 as compared to TNF‐α‐treated NPCs. NPC, nucleus pulposus cell; TNF‐α, tumor necrosis factor‐α [Color figure can be viewed at wileyonlinelibrary.com]

Gel zymography was performed in cell layer lysates for the detection and assessment of the gelatinases (MMP2/MMP9) expressed in the different treatment groups. It was able to detect both the MMP2 and MMP9 and their respective pro‐MMP2/MMP9 in the lysates (Figure 3A). However, the band intensities were almost the same in the lysates with insignificant variations amongst the treatment groups.

Nitrite production was significantly increased (*p* < 0.0001) following treatment of NPCs with TNF‐α at 24 h versus untreated control group (Figure 3B). U0126 dose‐dependently eliminated the TNF‐α‐mediated increase of nitrite at 0.5 µM (*p* = 0.0017) and to baseline levels at 5 µM (*p* < 0.0001).

### 3.4 Activation of ERK signaling

**Figure 4:**
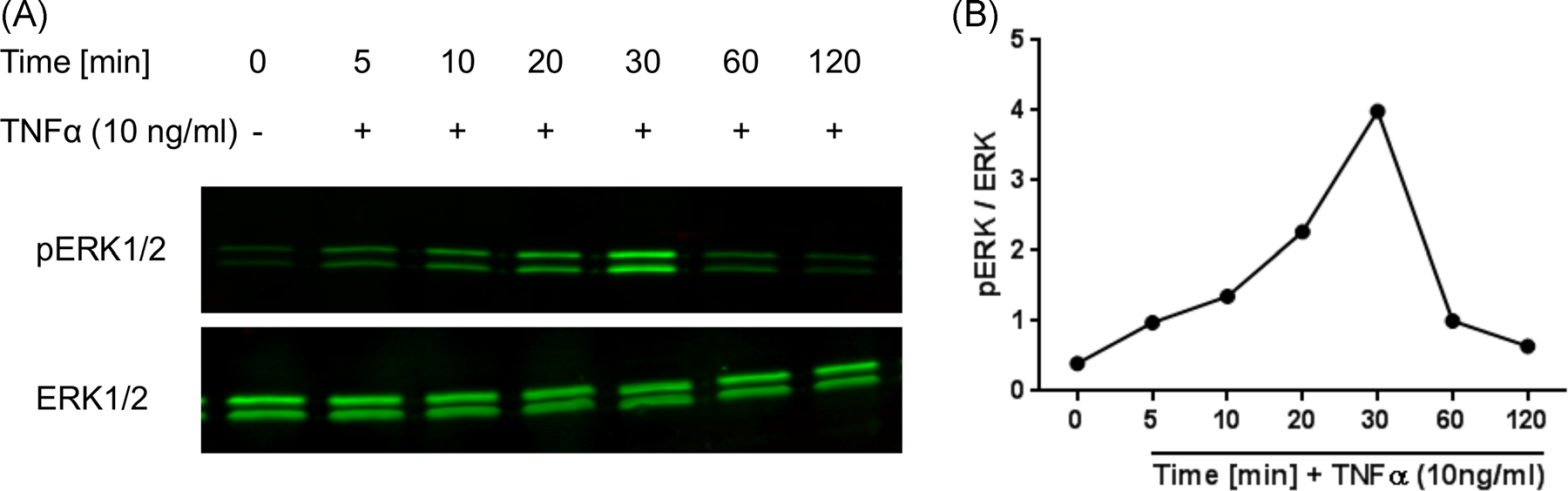
Western blot of phosphorylated ERK1/2 (p‐ERK) and their respective total ERK1/2 in NPCs after TNF‐α stimulation. (A) The NPCs were stimulated with TNF‐α (10 ng/ml) and the cells lysates were collected at different time points (minutes until 2 h) with control being time 0 min before stimulation. (B) p‐ERK and total ERK were determined using Western blot, the signal intensities from a representative experiment were quantified, and the p‐ERK were normalized to total ERK. ERK, extracellular signal‐regulated kinase; NPC, nucleus pulposus cell; TNF‐α, tumor necrosis factor‐α [Color figure can be viewed at wileyonlinelibrary.com]

**Figure 5:**
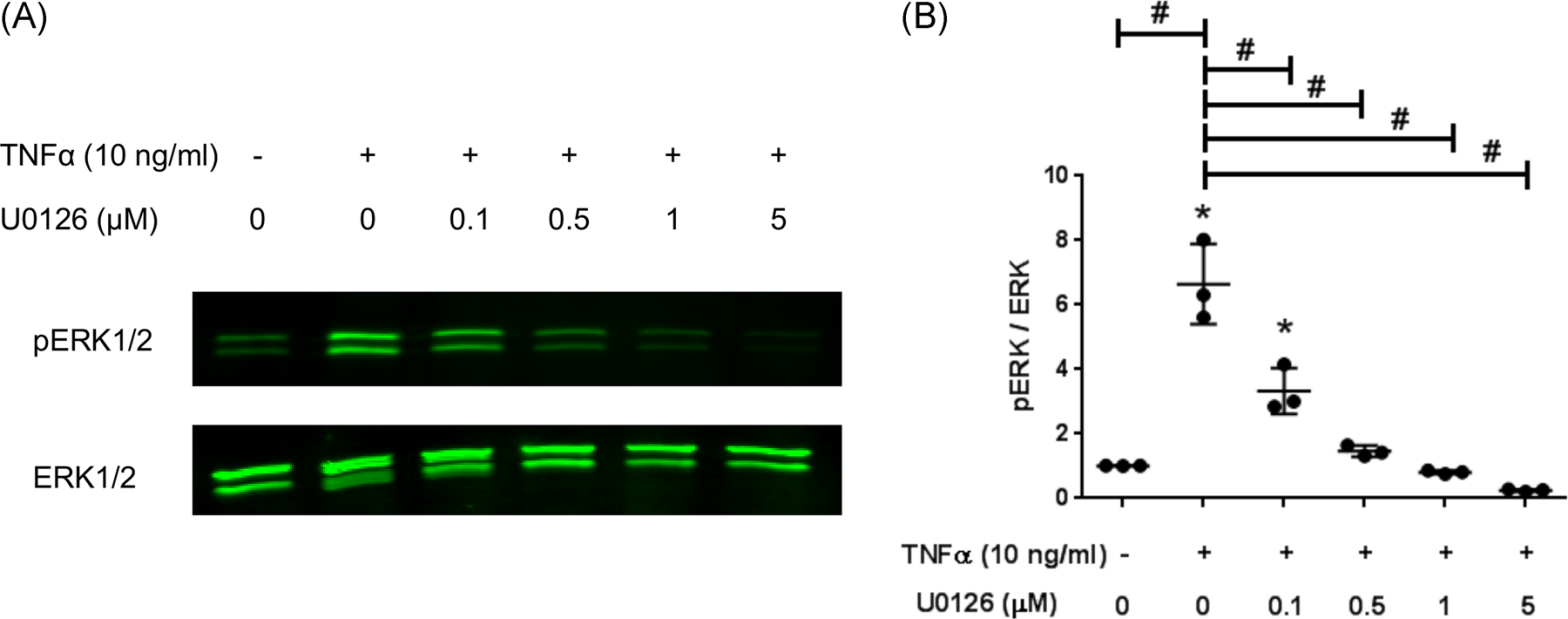
Western blot of phosphorylated ERK1/2 (p‐ERK) and their respective total ERK1/2 in TNF‐α‐stimulated NPCs with or without ERK inhibition. The ERK pathway in NPCs was blocked with U0126 (0.1, 0.5, 1, and 5 µM) for 1 h prior TNF‐α (10 ng/ml) treatment. (A) The cells were stimulated with TNF‐α and the cells lysates were collected for 30 min of treatments. The negative control being the unstimulated cells (Lane 1), the positive control being the TNF‐α‐alone treated NPCs (Lane 2), and the treatments being simultaneous TNF‐α and U0126‐treated samples (Lanes 3–6). The Western blot shown was from a representative experiment. (B) The p‐ERK1/2/ERK1/2 ratios were quantified in the different NPCs samples. U0126 abolished the TNF‐α‐mediated increase in pERK1/2/ERK1/2 in a dose‐dependent manner with results similar to untreated control at 0.5 µM (*p* > 0.999). *p < 0.05 as compared to untreated controls or ^#^ *p* < 0.05 as compared to TNF‐α‐treated NPCs. ERK, extracellular signal‐regulated kinase; NPC, nucleus pulposus cell; TNF‐α, tumor necrosis factor‐α [Color figure can be viewed at wileyonlinelibrary.com]

Western blots were carried out to assess the effects of TNF‐α and U0126 on the ERK mitogen‐dependent intracellular signaling. TNF‐α induced the ERK signaling pathway in human NPCs as demonstrated by the phosphorylation of ERK1/2 (Figure 4A). Notably, the peak ERK phosphorylation was achieved after 30 min of TNF‐α treatment in NPCs. The cytokine‐mediated activation of the ERK pathway was typically transient with a maximal TNF‐α stimulation obtained followed by a decline of p‐ERK dynamics after the first 30 min of TNF‐α treatment (Figure 4B). Further Western blots investigating the effect of U0126 on ERK pathway in TNF‐α‐stimulated NPCs were carried out at 30 min of cytokine exposure to benefit from the maximal TNF‐α stimulation effect. U0126 eliminated the TNF‐α‐mediated ERK phosphorylation in a dose‐dependent manner (Figure 5A). Cells treated with 0.5 µM U0126 along with TNF‐α resulted in phosphorylated ERK results comparable (*p* > 0.999) to the untreated control group (Figure 5B).

## 4 DISCUSSION

Current treatments to alleviate chronic low back pain are primarily surgical and have variable outcomes. For example, lumbar surgeries with and without spinal fusion have a 10%–40% failure rate, and similar results were obtained in patients that underwent a discectomy of herniated disc with recurrence after 2 years of surgery.^26^, ^27^, ^28^, ^29^ Innovative treatment strategies for IVD degeneration utilizing regenerative medicine or pharmacological inhibitors are urgently needed.

The present study investigated the contribution of ERK signaling in in vitro inflammation‐induced human NPCs by TNF‐α and whether ERK inhibition using selective mitogen‐activated protein kinase enzyme inhibitor can reverse the inflammatory phenotype of these cells. It was found that TNF‐α stimulation in NPCs induced a proinflammatory microenvironment of human IVD cells, in particular, NPCs, characterized by increased expression of important catabolic enzymes and proinflammatory mediators. Herein, the expression of matrix proteinases including MMP3 and MMP13 was upregulated in TNF‐α‐stimulated NPCs. MMPs are believed to be the major proteolytic enzymes responsible for ECM degradation in the IVD leading to disc degeneration.^30^ The expression of numerous metalloproteinases at the transcript and protein levels has been studied in several human IVD and experimental animal models, revealing catabolic changes and their mediation in the progression of IVD degeneration.^31^, ^32^, ^33^

This study further supports that the TNF‐α cytokine is a potent mediator of inflammatory response in the IVD, particularly in NPCs, and leads to disc degeneration through increased catabolism due to ECM degradation. Inflammatory processes exacerbated by TNF‐α and/or IL1β are believed to trigger disc degeneration and, in later stages, low back pain. For instance, surgical samples obtained from patients with a history of low back pain revealed higher levels of TNF‐α‐positive cells than autopsy from healthy controls.^11^ In addition, these intradiscal proinflammatory cytokines are implicated in the onset and progression of IVD degeneration and discogenic pain, and are produced by native IVD cells including NPCs and AFCs as well by infiltrating macrophages.^5^, ^9^, ^10^ Taken together, TNF‐α is an essential initiator of the proinflammatory environment in IVD tissue and cells that leads to the tissue ECM degradation and disorganization, and, therefore, to disc degeneration and back pain. The importance of TNF‐α in discogenic pain led to multiple clinical trials using TNF‐α inhibitors which resulted in mixed results,^34^, ^35^, ^36^ highlighting the need for further research studies.^12^ In particular, monoclonal antibodies (mAbs) against TNF‐α have shown promise for mitigating disc degeneration and relieving low back pain. Anti‐ TNF‐α treatment significantly decreases the concentration and activity of MMP1 and MMP3 in ex vivo IVD tissues isolated from patients with herniated discs.^37^ Despite the obvious benefit of TNF‐α mAbs, some patients do not respond to them and many will develop recurrent disease despite continuing dosing, which hampers the clinical use of these antibodies.^38^ In addition, Infliximab, a TNF‐α blocker and a chimeric immunoglobulin G1 antibody, did not appear to interfere with spontaneous resorption of disc herniation over a prolonged period based on magnetic resonance imaging diagnosis in a randomized controlled study.^39^ Therefore, further research to elucidate the mechanism by which inflammatory cascade is initiated through TNF‐α is required for targeted pharmacological treatment of IVD degeneration.

Within this study, we aimed to block the TNF‐α downstream signaling pathway by targeting the ERK from the MAPK family. First, we reproduced an in vitro inflammatory environment in NPCs and subsequently blocked the ERK pathway using U0126. The current study sought to elucidate the role of the ERK1/2 signaling pathway in a TNF‐α‐mediated catabolic environment in NPCs.

A resazurin red assay was used to examine the possibility of interfering of the ERK inhibitor with NPCs metabolic activity. It was found that U0126 reduced metabolically active cells at large concentrations, translated by a dose‐dependent reduction of cells’ metabolism with increasing U0126 molarity. However, U0126 did not decrease the number of viable cells as detected by labeling the cells with calcein‐AM and ethidium homodimer‐1. This finding further supports the critical role of MAPK, in particular, the ERK1/2 pathway, in the regulation of mammalian cell proliferation as previously documented.^40^

Blocking the ERK pathway downregulated the TNF‐α‐induced expression of MMP3 and MMP13 mRNA levels in human NPCs. It was previously demonstrated that CCAAT/enhancer‐binding protein β (C/EBPβ) in the TNF‐α promoter region was suppressed in the presence of an ERK inhibitor PD98059 and the p38‐MAPK inhibitor SB202190, but not the c‐Jun N‐terminal kinase (JNK) inhibitor SP600125 in rat NPCs.^41^ In addition, the C/EBPβ and MMP13 expression was colocalized in chondrocytes in inflammatory arthritic patients^42^ and proinflammatory cytokines such as IL1β and TNF‐α bind to MMP3 and MMP13 promoter regions and stimulate their expression.^42^, ^43^ Similarly, treatment of rat NPCs with ERK1/2 inhibitors (PD98059 and U0126) abolished the antagonistic effect of TGF‐β1 on TNF‐α mediated MMP3 catabolic response,^44^ which further supports our finding on the implication of ERK pathway in inflammatory human NPCs. Taken together, TNF‐α induces an inflammatory cascade in mammalian cells, in particular, NPCs, by upregulation and modulation of MMP family members, such as MMP3/MMP13 through the ERK1/2 pathway, and inhibition of ERK signaling can reverse this catabolic effect. Therefore, ERK1/2 can be considered as a downstream signaling pathway of TNF‐α and MAPK might be a target for the increased MMP enzymatic activity. Nevertheless, the enzymatic activities mediated by the gelatinases MMP2/MMP9 were visible on gel zymography in NPCs cell layer lysates, it was not possible to detect significant variations of MMP2/MMP9 following treatment of NPCs with TNF‐α. This could be explained by an inappropriate model for the detection of MMP2/MMP9, as the intracellular gelatinases MMPs are normally secreted in the extracellular compartment for a variety of cell lineages.^45^, ^46^, ^47^ Therefore, it is more relevant to assess the incorporated gelatinases in culture supernatant rather than within the cells lysates^48^ as also detected previously for IVD cells.^49^ TNF‐α‐induced NPCs resulted in increased nitrate release in the cell culture medium which is in line with a recent study of TNF‐α injection in ex vivo IVD organ culture model^21^ and ERK inhibition abolished the nitrate production.

The activation of an inflammatory microenvironment through stimulation of NPCs with TNF‐α resulted in an increased ADAMTS5 expression compared to controls. Blocking the ERK1/2 pathway with U0126, however, did not alter the expression of ADAMTS5. This observation might be explained by a differentiation regulation of aggrecanase‐mediated proteoglycan degradation, including ADAMTS4‐5 which is mediated through NF‐kB activation and not ERK1/2 in bovine NPCs.^50^ Similarly, it was suggested that ADAMTS4 expression and promoter activity increased in NPCs following TNF‐α and IL‐1β treatments^51^ and treatment of the cells with NF‐kB inhibitor abolished this inductive effect of the cytokines on ADAMTS4 mRNA and protein expression. This further supports that modulation of ADAMTS5 in NPCs is mediated by NF‐kB and could explain our observation on the role of ERK1/2 in ADAMTS5 expression.

The results of this study revealed a significant increase of KRT19 in inflammatory NPCs that were treated with U0126, and insignificant random changes in transcript levels of the NP‐specific markers including COL2 and ACAN. In addition, a minimal variation in the expression of anabolic genes such as IGF1 was observed, suggesting a nominal effect on the NPCs phenotype with regard to anabolic metabolism following the inhibition of the ERK pathway in inflammatory cells. Similarly, Wei et al.^17^ observed an imbalance between anabolic and catabolic events in rat AFCs activated with IL1, and ERK inhibition significantly blocked the catabolic and inflammatory effects of IL1 in AFCs.

Within this study, we provided evidence that the ERK1/2 pathway triggers TNF‐α‐mediated inflammation in human NPCs and leads to increased proinflammatory mediators, such as MMPs, and some decreased anabolic genes characteristic of degenerated IVD cells. A Western blot assay conveyed that the ERK1/2 pathway was modulated through TNF‐α, while simultaneous treatment of NPCs with TNF‐α and U0126 abolished this effect. This reveals that U0126 is a specific inhibitor of the ERK1/2 pathway.

Unfortunately, there are some limitations of the current study. First, due to the difficulty and availability in obtaining healthy and nondegenerated human disc tissues samples, the results were limited to gene expression analysis of some anabolic and catabolic genes, and we did not investigate their protein levels in more detail. Second, we did not address the contribution of other MAPKs, such as p38‐MAPK and JNK, in inflammation, although some pilot data was generated. In addition, we used in vitro monolayer cultures of NPCs, which might differ from three‐dimensional cultures such as cells‐seeded scaffolds or ex vivo IVD samples. Furthermore, the combined effects of inflammatory mediators and biomechanical stimuli were not addressed in this study; however, future investigations aim to explore this.

In conclusion, the current study provides evidence that the ERK pathway is implicated in NPCs inflammatory processes and ERK1/2 inhibition could provide some protection against the adverse effects of TNF‐α in the IVD.

## AUTHOR CONTRIBUTIONS

Adel Tekari designed and conducted the experiments, collected the data, and wrote the manuscript draft. Alessandro Marazza performed additional experiments and assisted in statistical analysis. Katherine Crump and Paola Bermudez‐Lekerika conducted further experiments and critically revised the manuscript. Benjamin Gantenbein prepared the research plan, conducted additional experiments, provided the funding, and reviewed the manuscript. All the authors read and approved the final manuscript.

## CONFLICTS OF INTEREST

The authors declare no conflicts of interest.
